# Genetic-Algorithm-Based Inverse Optimization Identification Method for Hot-Temperature Constitutive Model Parameters of Ti6Al4V Alloy

**DOI:** 10.3390/ma16134726

**Published:** 2023-06-29

**Authors:** Xuewen Chen, Zhiyi Su, Jiawei Sun, Zhen Yang, Bo Zhang, Zheng Zhou

**Affiliations:** School of Materials Science and Engineering, Henan University of Science and Technology, 263 Kaiyuan Avenue, Luoyang 471023, China; 210321020235@stu.haust.edu.cn (Z.S.); 210321020208@stu.haust.edu.cn (J.S.); 210321020250@stu.haust.edu.cn (Z.Y.); 220320020200@stu.haust.edu.cn (B.Z.); 220320020243@stu.haust.edu.cn (Z.Z.)

**Keywords:** inverse optimization, genetic algorithm, Ti6Al4V alloy, strain compensation, coupling effect

## Abstract

A precise constitutive model is the foundation and key to finite element simulation in material volume forming and the optimization of the hot working process. Hence, to build a precise constitutive model, a method based on a genetic algorithm (GA) for the inverse optimization identification of parameters is presented in this paper. The idea of this method is to continuously adjust the model parameters through GA until the objective function reaches the minimum value. In this study, hot compression experiments were performed on the Gleeble-1500D thermal simulator at temperatures ranging from 800 °C to 1000 °C and strain rates of 0.01 s^−1^ to 1 s^−1^. The Arrhenius-type (A-T) model considering strain compensation and the Johnson–Cook (JC) model considering the coupling effects of strain, temperature and strain rate were constructed, respectively, by using the regression method and the parameter inverse optimization identification method. For the purposes of comparing and verifying the reliability of the predictions of the two established constitutive models, the correlation coefficient (R), average absolute relative error (AARE), and relative error (RE) were adopted. The results show that both the optimized A-T model and the optimized JC model have high prediction accuracy. Compared to the optimized JC model, the optimized A-T model demonstrated a higher correlation coefficient, by 0.003, and a lower average absolute relative error, by 1.43%. Furthermore, the relative error distribution of the optimized A-T model was found to be more concentrated than that of the optimized JC model. These results suggest that the A-T model is more appropriate than the JC model for characterizing the high-temperature deformation behavior of Ti6Al4V alloy.

## 1. Introduction

Because of the outstanding characteristics of high specific strength, corrosion resistance, and biocompatibility, titanium alloys have found extensive applications in various fields such as aerospace, the chemical industry, automobiles, and medical implants [1,2,3]. Among all titanium alloys, Ti6Al4V alloy is the most frequently used high-strength duplex (α+β) titanium alloy [4]. At room temperature, the Ti6Al4V alloy has poor formability due to its densely packed hexagonal crystal structure, which has only three slip systems. This makes it difficult to produce complex-shaped parts [5]. Thus, the hot formation of Ti6Al4V alloy has always been the focus of research by scholars [6,7]. Different hot deformation conditions will cause different microstructure evolutions of the Ti6Al4V alloy during hot forming, resulting in a highly nonlinear relationship between stress and temperature, strain rate, and strain at the macroscopic level [8]. Therefore, studying the hot deformation behavior of metals and establishing constitutive models that precisely characterize the metal flow behavior are highly important for process designers to reasonably design hot working process parameters, optimize the hot forming process of materials, and ultimately improve the overall mechanical properties of products.

Currently, there are three broad categories of the constitutive models: phenomenological, physics-based, and artificial neural network (ANN) models [9,10,11]. The phenomenological constitutive model has a simple form, fewer parameters, and can be easily integrated into the finite element software. The common ones are the Johnson–Cook (JC) model [12], the Arrhenius-type (A-T) model [13], the Fields–Bachofen (FB) model [14], the Voce–Kocks model [15], and the Hansel–Spittel model [16]. The most widely used constitutive models of this type are the JC model and the A-T model. The traditional JC model neglected the mutual influence of temperature softening, strain hardening, and strain rate hardening, resulting in the low prediction accuracy of the model. Lin et al. [17] established a modified JC model. The initial yield and strain hardening parts, and the coupling effects of temperature and strain rate on flow stress, were accounted for in the model. He [18] and Long [19] predicted the high-temperature deformation behavior of 20CrMo alloy steel and Al-Cu-Li alloy, respectively, through this modified model. The parameters of the original A-T model are often determined based on experimental data at a given strain or peak stress. The application of this model is limited. Lin et al. [20] considered the impact of strain on material parameters and established an A-T model for 42CrMo alloy steel considering strain compensation. Since then, this model has been employed by numerous researchers to successfully reproduce the high-temperature deformation behavior of various materials such as the 2219-O aluminum alloy, the AZ91 magnesium alloy, and the GH4169 alloy [13,21,22].

Physics-based constitutive models are mainly derived from theories of thermodynamics, thermally activated dislocation motion, and slip dynamics [11]; for example, the Zerlil–Armstrong (ZA) model [23] and the Voyiadjis–Almasri (VA) model [24], etc. Owing to the complex microstructure evolution of the Ti6Al4V alloy and the difficulty of using a physics-based constitutive model in finite element simulation software, the application of such constitutive models to Ti6Al4V alloys has been relatively limited, with few studies conducted to date [14,25]. With the swift development of artificial intelligence technology in recent years, the ANN has been considered to be the optimal method for describing and solving highly nonlinear problems [26]. Although the ANN constitutive model has high prediction accuracy, the accuracy of its prediction results is limited to the given parameter range of the learning samples. Wen DX et al. [27] established an A-T model through the NM simplex method and compared the prediction results with the ANN model. The results revealed that the generalization ability of the latter was not as good as that of the former. Therefore, this paper does not consider the ANN constitutive model of the Ti6Al4V alloy.

It is impossible for a single constitutive model to be suitable for all materials. Therefore, numerous scholars have conducted comparative studies on the constitutive relationships of different materials. Li T et al. [28] established the JC, A-T and ZA models of the SnSbCu alloy and compared the prediction accuracy of the three constitutive models. Abbasi-Bani [29] established the JC and A-T constitutive models of the Mg-6Al-1Zn alloy, and found that the former model could not correctly predict the high-temperature deformation behavior of the alloy. However, there are relatively few studies on the comparison of constitutive models for the Ti6Al4V alloy. To find out the most suitable constitutive model for the Ti6Al4V alloy, the A-T model, considering the strain compensation, and the JC model, considering the coupling effects of temperature, strain rate and strain, were chosen in this paper. The two models were compared and the model with the best predictive performance was selected.

In addition, the accurate identification of model parameters is also an important factor affecting the prediction accuracy of alloy flow behavior [30]. The parameters of the constitutive model are often obtained using the regression method [31,32].

As in the Hansel–Spittel constitutive model mentioned above,

(1) $$\sigma =A\cdot e^{m_{1}T}\cdot \varepsilon^{m_{2}}\cdot {\dot{\varepsilon }}^{m_{3}}\cdot e^{\frac{m_{4}}{\varepsilon }}\cdot {\left(1+\varepsilon \right)}^{m_{5}T}\cdot e^{m_{7}\varepsilon }\cdot {\dot{\varepsilon }}^{m_{8}T}\cdot T^{m_{9}}$$

For the solution of parameters $m_{3}$ and $m_{8}$, the temperature and strain are firstly controlled to be constant. Then, the terms of the above formula for temperature and strain are all constants, which are set as K. Equation (1) can be changed to:

(2) $$\sigma ={\dot{\varepsilon }}^{m_{3}+m_{8}T}\cdot K$$

Equation (2) can be transformed by taking the natural logarithm of both sides:

(3) $$ln\sigma =(m_{3}+m_{8}T)ln\dot{\varepsilon }+lnK$$

From Equation (3) we can obtain:

(4) $$m_{3}+m_{8}T=\frac{\partial ln\sigma }{\partial ln\dot{\varepsilon }}$$

The average slope of the fitted lines based on the relationship between lnσ and $ln\dot{\varepsilon }$ can be used to determine the value of $m_{3}+m_{8}T$. Then, the relationship between $m_{3}+m_{8}T$ and T can be linearly fitted. It can be clearly seen that the value of $m_{8}$ is given by the slope of the fitted line, while the value of $m_{3}$ is given by the intercept. Likewise, the remaining parameters of the Hansel–Spittel constitutive model can also be derived using this approach, if the maximum and minimum values of the slope of the fitted straight lines are quite different. At this time, the selected average value will cause a large error in $m_{3}+m_{8}T$. The same goes for other parameters. Finally, the accuracy of the model cannot be guaranteed. The Hansel–Spittel model established by Chadha [31] had a maximum error of 14% between the predicted values and experimental values, and at low strain rates the model’s ability to predict the alloy’s softening behavior was limited. Consequently, the constitutive models established through the regression method often fail to precisely predict the flow behavior of alloys.

In recent years, the inverse analysis method has been widely used in the identification process of parameters in various industries [33,34,35,36]. For example, Levasseur S [33] used the inverse analysis method to identify the parameters of the soil constitutive model. Gajewski T [34] quickly calibrated a complete set of parameters for concrete using an inverse analysis method. Among them, the genetic algorithm (GA) is a widely used global optimization algorithm. It seeks the optimal solution to a problem by imitating the phenomena of replication, crossover, and mutation in natural selection and heredity. The main method of using GA to solve the optimal solution of parameters is through iterative operation. Some optimization algorithms are prone to become trapped in local minima and can exhibit the “dead loop” phenomenon, but GA can overcome this shortcoming very well. Compared with traditional optimization methods (enumeration, heuristic, etc.), GA has less calculation time and characteristics of good convergence and robustness. Both Wu [37] and Chen W [38] employed the genetic algorithm (GA) to perform inverse optimization of the material parameters in the A-T model. It was found that the A-T model’s forecasting accuracy greatly increased. Through investigation, it was found that there are few studies on the optimization of constitutive model parameters for the Ti6Al4V alloy. Therefore, a parameter inverse optimization identification method based on GA to develop the constitutive model of Ti6Al4V alloy was chosen in this paper.

This paper aims to develop a precise constitutive model and lay the foundation for the subsequent reasonable design of Ti6Al4V thermal processing parameters and finite element simulation. The Ti6Al4V alloy’s hot deformation characteristics were analyzed using the Gleeble-1500D thermal simulator. In order to accurately identify the parameters of the constitutive model, a parameter inverse optimization identification method based on the genetic algorithm was proposed in this paper. The objective function was the accumulated error between the stress calculated by the constitutive model and the experimental stress. This method iteratively adjusts the parameters of the constitutive model through the genetic algorithm until the objective function reaches the minimum value, and the output parameters are the optimal solution of the parameters of the constitutive model. Finally, the prediction accuracy of the two models was quantitatively evaluated using standard statistical parameters, and the constitutive model with the highest prediction accuracy was selected.

## 2. Experimental Method and Result Analysis

### 2.1. Experimental Method

**Figure 1:**
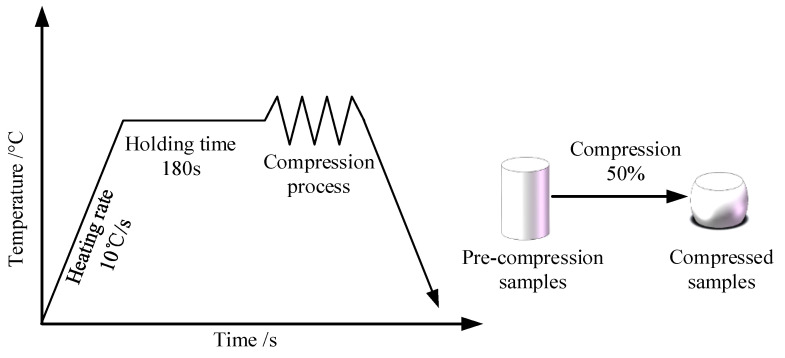
Experimental procedure for studying Ti6Al4V alloy.

The chemical composition of the Ti6Al4V alloy can be seen in Table 1. To investigate the hot deformation characteristics of the Ti6Al4V alloy and establish its constitutive model, thermal compression tests were carried out on the Gleeble-1500D thermal simulator. The size of the compression samples was ϕ8 mm × 12 mm. To make the deformation of the samples uniform and mitigate or eliminate the impact of the frictional forces on the experimental results, graphite was uniformly applied to both ends of the cylinder in contact with the indenter of the testing machine. The deformation temperatures were 800 °C, 850 °C, 900 °C, 950 °C, and 1000 °C; the strain rates were 0.01 s^−1^, 0.1 s^−1^, and 1 s^−1^. The thermal compression experiment process is shown in Figure 1. Firstly, a heating rate of 10 °C per second was applied to each cylindrical sample. After reaching the predetermined temperature, it was kept warm for 180 s to remove the internal temperature gradient of the samples. Lastly, compression tests were conducted and each sample was compressed by 50%.

### 2.2. Analysis of Experimental Results of the Ti6Al4V Alloy

**Figure 2:**
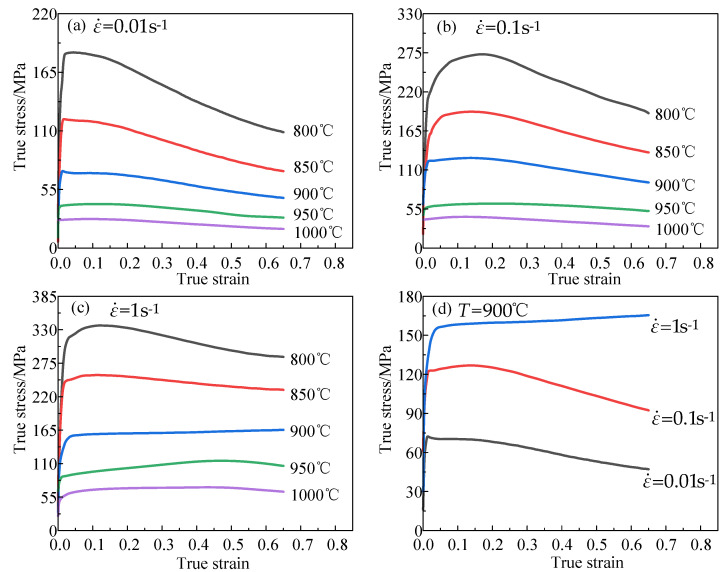
True stress–strain curves of Ti6Al4V alloy in various situations. (**a**) $\dot{\varepsilon }$ = 0.01 s^−1^; (**b**) $\dot{\varepsilon }$ = 0.1 s^−1^; (**c**) $\dot{\varepsilon }$ = 1 s^−1^; (**d**) T = 900 °C.

The true stress–strain curves of the Ti6Al4V alloy under different deformation conditions can be observed in Figure 2. Holding the strain rate constant, an increase in temperature resulted in a decrease in flow stress. From Figure 2c, it can be observed that the material’s peak stress decreased from 336.95 MPa at 800 °C to 71.09 MPa at 1000 °C. This was because the thermal activation energy of the alloy increased with an increase in deformation temperature, leading to a more pronounced softening effect. At a constant temperature, the flow stress rose with an increase in the strain rate. According to Figure 2d, the peak stress of the material increased from 72.26 MPa at 0.01 s^−1^ to 165.47 MPa at 1 s^−1^.This is because the strain rate was too fast, and dislocations accumulated rapidly, resulting in the obvious work hardening effect of the Ti6Al4V alloy.

In addition, since the temperature was below 850 °C, the main microstructure of the Ti6Al4V alloy was α phase. The α phase is a hexagonal close-packed structure with low stacking fault energy, which makes it relatively easy for dislocations to move in the crystal, and dynamic recrystallization occurs easily. Therefore, the dynamic recrystallization softening phenomenon could be clearly observed at 800–900 °C. At the 800 °C/0.1 s^−1^ deformation condition, the flow stress decreased by 82.98 MPa from its peak value, as shown in Figure 2b. The dynamic recovery phenomenon appeared under the deformation condition of 900 °C/1 s^−1^, as shown in Figure 2c. This is because the recrystallization time was relatively short at high strain rates, so the stress rose slowly to 165.47 MPa after quickly reaching 152.03 MPa.

The α phase will gradually transform into the β phase above 850 °C. In contrast to the α phase, the β phase is a body-centered cubic structure with high stacking fault energy and many slip systems, resulting in insufficient deformation energy to provide the driving force for dynamic recrystallization. Therefore, above 950 °C, dynamic recovery is the main softening mechanism of the alloy, and the stress has no obvious downward trend.

## 3. Two Modified Constitutive Models of the Ti6Al4V Alloy and the Identification Method of Parameter Inverse Optimization

### 3.1. Parameter Inverse Optimization Identification Method Based on GA

**Figure 3:**
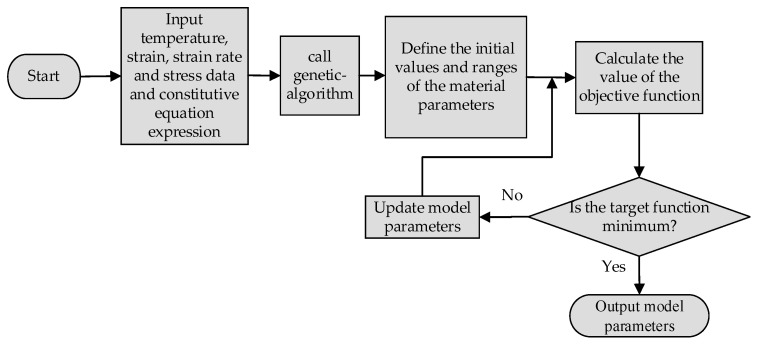
Flowchart of parameter inverse optimization identification method.

The idea of the parameter inverse optimization identification method is to continuously adjust the parameters of the constitutive model through GA. When the objective function approaches zero infinitely, the output parameters are the optimal solution. Figure 3 shows the logical flowchart of this method. Firstly, input the data obtained from thermal compression experiments and the expression for the constitutive model. After that, the GA is invoked. Finally, set the initial value and iteration range of the constitutive model parameters.

The material parameters obtained by the regression method were set as the initial values of the GA iteration. Section 3.2 and Section 3.3 describe how to determine constitutive model parameters using the regression method. The setting of the model parameters’ iteration range refers to the initial values. In this paper, the cumulative error between the stress predicted by the Ti6Al4V alloy constitutive model and the experimentally measured stress is set as the objective function, as shown in Formula (5):

(5) $$O(f)=\frac{\sum_{i}^{n}{\left(\sigma_{i}{}^{exp}-\sigma_{i}{}^{cal}\right)}^{2}}{\sum_{i}^{n}{\left(\sigma_{i}{}^{exp}\right)}^{2}}$$

where $\sigma_{i}{}^{exp}$ is the i-th stress measured experimentally and $\sigma_{i}{}^{cal}$ is the stress of the i-th data calculated by the constitutive model.

### 3.2. Establishment of A-T Constitutive Model for the Ti6Al4V Alloy

An exponential equation that includes the Zener–Hollomon (Z) parameter is commonly used to describe the effect of temperature and strain rate on the thermal deformation behavior of metal materials [39]:

(6) $$Z=\dot{\varepsilon }\exp\left(\frac{Q}{RT}\right)$$

Furthermore, the Z parameter can be described by various functions in distinct stress ranges [40]. The expressions for each range are provided below:

(7) Z=AF(σ)

(8) $$F(\sigma )=\left\{\begin{array}{cc} \sigma^{n\prime } & \alpha \sigma <0.8\\ \exp(\beta \sigma ) & \alpha \sigma >1.2\\ \left[\sinh(\alpha \sigma {)}^{n}\right] & for\;all\;\sigma \end{array}\right.$$

The characters in Formulas (6)–(8) are explained in Table 2. The solution formula of flow stress can be obtained by combining Equations (6) and (7) and the third equation in Equation (8), and the expression is as follows:

(9) $$\sigma =\frac{1}{\alpha }\ln\left({\left(\frac{Z}{A}\right)}^{1/n}+{\left({\left(\frac{Z}{A}\right)}^{2/n}+1\right)}^{1/2}\right)$$

Furthermore, combining Equations (6)–(8), the following relationship can be derived:

(10) $$\left\{\begin{array}{l} \dot{\varepsilon }=A\sigma^{n\prime }\exp(-\frac{Q}{RT})\\ \dot{\varepsilon }=A\exp(\beta \sigma )\exp(-\frac{Q}{RT})\\ \dot{\varepsilon }=A[\sinh(\alpha \sigma ){]}^{n}\exp\left(-\frac{Q}{RT}\right) \end{array}\right.$$

The natural logarithm of both sides of the three equations in Formula (10) can be taken to yield:

(11) $$\left\{\begin{array}{l} \ln\sigma =\frac{\ln\dot{\varepsilon }}{n\prime }-\frac{\ln B}{n\prime }\\ \sigma =\frac{\ln\dot{\varepsilon }}{\beta }-\frac{\ln C}{\beta }\\ \ln(\sinh(\alpha \sigma ))=\frac{Q}{nRT}-\frac{\ln A}{n}+\frac{\ln\dot{\varepsilon }}{n} \end{array}\right.$$

In the equations, *B* and *C* are material parameters.

The impact of strain was factored in when determining the values of material parameters. In this paper, the relationship between strain and material parameters was constructed using polynomial regression. The material parameters were determined using experimental data within the true strain range of 0.05–0.65. The sampling interval of strain was 0.05. Here, the process for determining the material parameters is illustrated using the example of a strain of 0.1.

**Figure 4:**
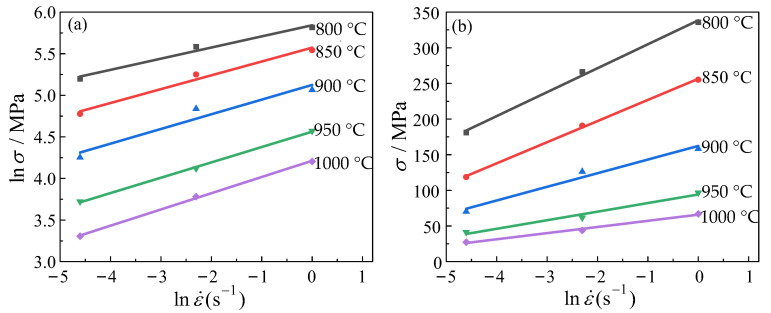
The relationship between the strain rate and the stress under different conditions: (**a**) $\ln\sigma -\ln\dot{\varepsilon }$; (**b**) $\sigma -\ln\dot{\varepsilon }$.

When the strain is 0.1, the experimental data are substituted into the first two equations of Equation (11). The material parameters *β* and n′ can be determined using a linear regression approach. In Figure 4a,b, were parameters are determined by computing the inverse of the slope of the fitted lines. The data analysis revealed that the two parameters had average values of 0.062997 MPa^−1^ and 5.938013, respectively. The material parameter α could be derived as 0.010609 MPa^−1^ using the equation α=β/n′.

**Figure 5:**
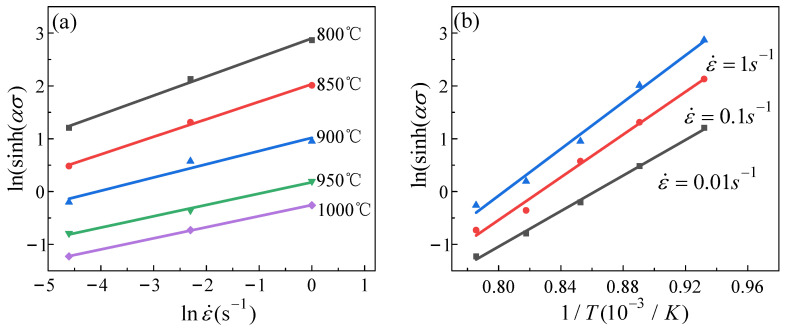
The relationship between strain rate, deformation temperature, and true stress under different conditions: (**a**) $\ln(\sinh(\alpha \sigma ))-\ln\dot{\varepsilon }$; (**b**) ln(sinh(ασ))−1/T.

Similarly, using the results of fitting straight lines in Figure 5a,b, the parameters *n*, *Q*, and lnA were calculated to have values of 3.839814, 630,040.1 J·mol^−1^, and 60.44771 s^−1^, respectively.

Likewise, parameters under other strains were determined in the same way. The results can be found in Table 3.

**Figure 6:**
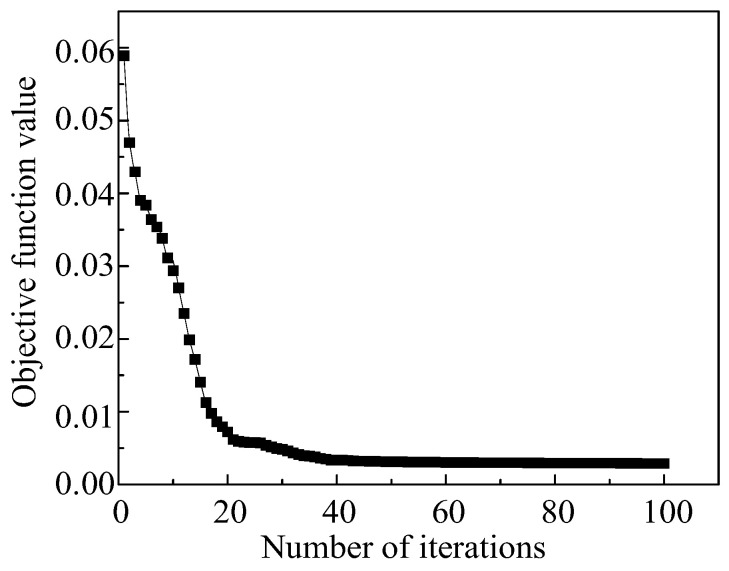
The optimization curve of the objective function of the A-T model when the strain is 0.1.

Using the parameter values at 0.1 strain in Table 3 as the initial values for the GA iteration and executing the flowchart in Section 3.1, under this strain, the change of the objective function value of the A-T model with the number of iterations is shown in Figure 6. As the iterations proceeded, the objective function value eventually converged to around 0.00289. The output of the logic diagram was the optimal solution of the constitutive model parameters under the strain. Similarly, using the same method, the optimal solutions of material parameters under other strains are shown in Table 4.

To improve the accuracy of polynomial regression, sixth-order polynomial regression was performed on the material parameters and true strain:

(12) $$\begin{array}{c} \alpha (\varepsilon )=B_{0}+B_{1}\varepsilon +B_{2}\varepsilon^{2}+B_{3}\varepsilon^{3}+B_{4}\varepsilon^{4}+B_{5}\varepsilon^{5}+B_{6}\varepsilon^{6}\\ n(\varepsilon )=C_{0}+C_{1}\varepsilon +C_{2}\varepsilon^{2}+C_{3}\varepsilon^{3}+C_{4}\varepsilon^{4}+C_{5}\varepsilon^{5}+C_{6}\varepsilon^{6}\\ Q(\varepsilon )=D_{0}+D_{1}\varepsilon +D_{2}\varepsilon^{2}+D_{3}\varepsilon^{3}+D_{4}\varepsilon^{4}+D_{5}\varepsilon^{5}+D_{6}\varepsilon^{6}\\ \ln A(\varepsilon )=E_{0}+E_{1}\varepsilon +E_{2}\varepsilon^{2}+E_{3}\varepsilon^{3}+E_{4}\varepsilon^{4}+E_{5}\varepsilon^{5}+E_{6}\varepsilon^{6} \end{array}$$

**Figure 7:**
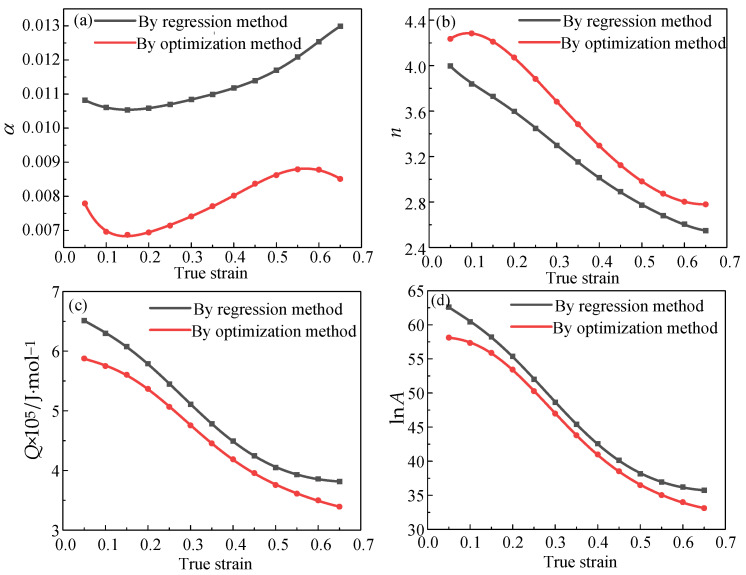
The variation of A-T model parameters with true strain. (**a**) α-ε; (**b**) n-ε; (**c**) Q-ε; (**d**) lnA-ε.

The relationship between true strain and material parameters is depicted in Figure 7. The four parameters changed greatly after optimization. Table 5 and Table 6 are the sixth-order polynomial coefficient values of the unoptimized A-T model and the optimized A-T model, respectively.

### 3.3. Establishment of the JC Constitutive Model for the Ti6Al4V Alloy

Apart from the A-T model, the JC model is also extensively employed during metal forming processes owing to its few parameters and simple fitting process [41]. In this paper, a modified JC constitutive model is proposed. Its expression is:

(13) $$\left\{\begin{array}{l} \sigma =A(\varepsilon )\cdot \left(1+B(\varepsilon )\ln{\dot{\varepsilon }}^{*}\right)\cdot \exp\left(\left(C(\varepsilon )+D(\varepsilon )\ln{\dot{\varepsilon }}^{*}\right)T^{*}\right)\\ \begin{array}{c} A(\varepsilon )=A_{0}+A_{1}\varepsilon +A_{2}\varepsilon^{2}+A_{3}\varepsilon^{3}\\ B(\varepsilon )=B_{0}+B_{1}\varepsilon +B_{2}\varepsilon^{2}+B_{3}\varepsilon^{3}\\ C(\varepsilon )=C_{0}+C_{1}\varepsilon +C_{2}\varepsilon^{2}+C_{3}\varepsilon^{3}\\ D(\varepsilon )=D_{0}+D_{1}\varepsilon +D_{2}\varepsilon^{2}+D_{3}\varepsilon^{3} \end{array} \end{array}\right.$$

The meanings expressed by each character in Formula (13) are listed in Table 7. In this paper, ${\dot{\varepsilon }}_{ref}$ is equivalent to 0.01 s^−1^ and $T_{ref}$ is denoted as 800 °C. The melting point of the Ti6Al4V alloy is 1660 °C [42].

**Figure 8:**
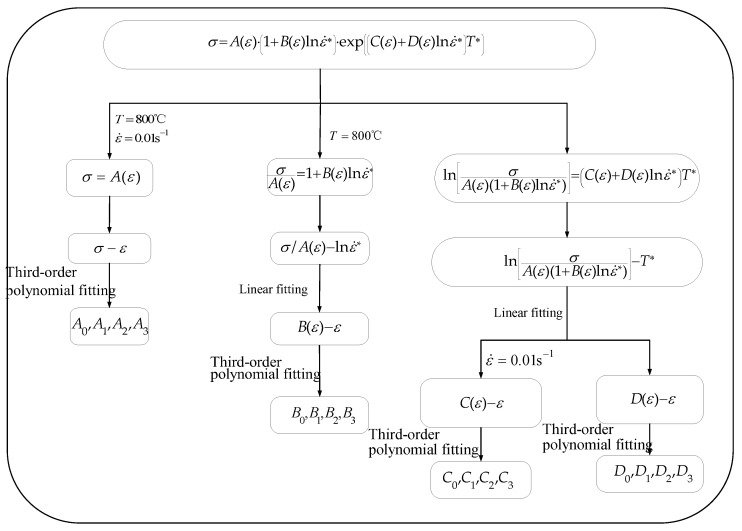
The flowchart for solving the parameters of the JC model through the regression method.

The flowchart for determining the material parameters of the JC model is presented in Figure 8. Firstly, under the condition of 800 °C/0.01 s^−1^, the relationship between ε and σ is fitted by a polynomial. The polynomial coefficients correspond to the values of parameters $A_{0}$, $A_{1}$, $A_{2}$, and $A_{3}$, consecutively.

Then, the relationship between σ/A(ε) and $\ln{\dot{\varepsilon }}^{*}$ was fitted linearly at 800 °C, and the values of B(ε) at different strains could be determined by the slopes of the fitted lines. Then, fit the relationship between B(ε) and ε through the third-order polynomial. Similar to the previous scenario, the coefficients of the third-order polynomial are indicative of the values of parameters $B_{0}$, $B_{1}$, $B_{2}$, and $B_{3}$, in that order.

Finally, a linear fit was made to the relationship between $\ln\frac{\sigma }{A(\varepsilon )(1+B(\varepsilon )\ln{\dot{\varepsilon }}^{*})}$ and $T^{{}^{*}}$. The values of $C(\varepsilon )+D(\varepsilon )\ln{\dot{\varepsilon }}^{*}$ corresponded to the slopes of the fitted lines. When the strain rate was fixed at 0.1 s^−1^, determining the value of parameters C(ε) at various strains was a simple task, and calculating parameters D(ε) was also possible. Likewise, by employing identical third-order polynomial regression, the parameters $C_{0}-C_{3}$ and $D_{0}-D_{3}$ could be determined.

**Figure 9:**
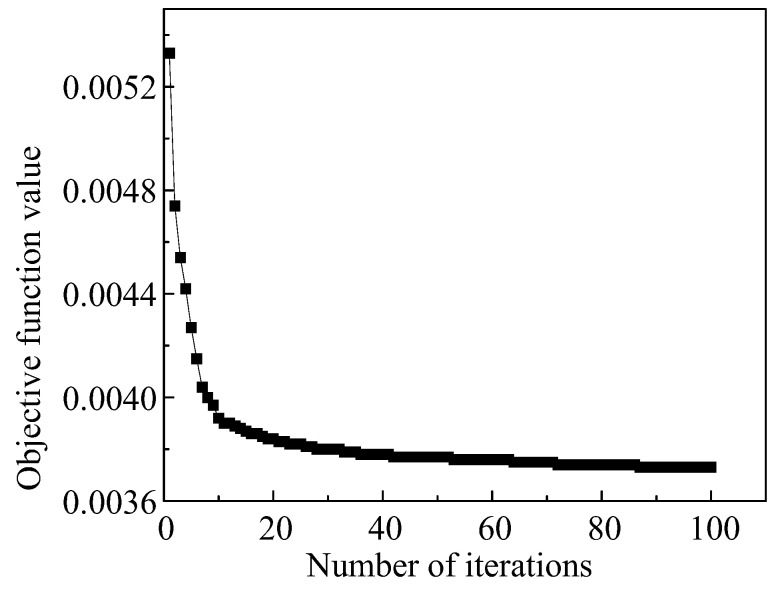
Iteration curves for the objective function values of the JC model.

In summary, the JC model’s parameters are listed in Table 8. These parameters were employed as the initial values for the GA iteration. Figure 9 is the optimization curve of the objective function of the JC model. The value of the objective function gradually decreased with the number of iterations, and finally tended to be around 0.00373. At this stage, the output results represent the optimal solution for the JC model parameters, as listed in Table 9.

## 4. Verification and Comparison of Prediction Accuracy of Two Constitutive Models for the Ti6Al4V Alloy

**Figure 10:**
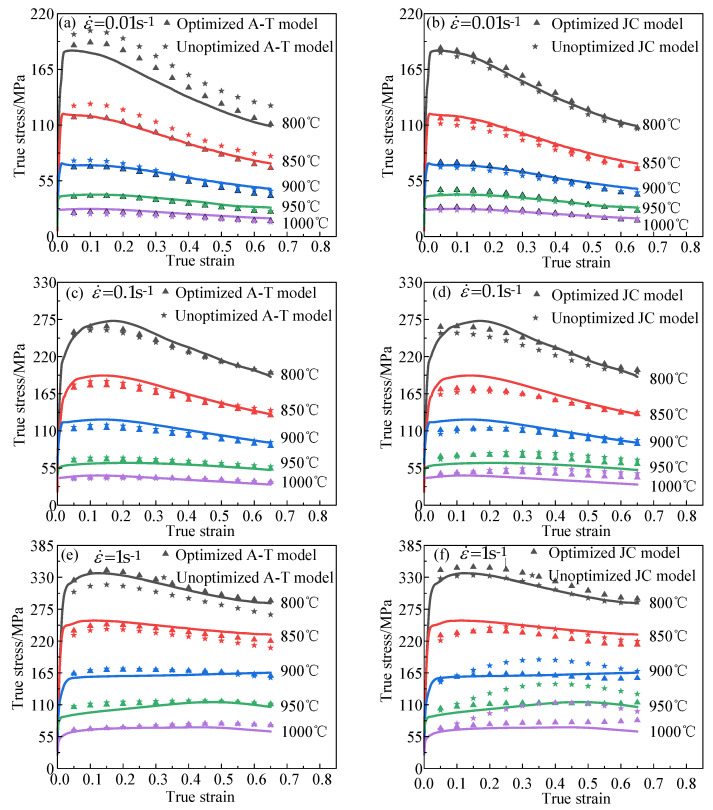
Comparison between the stress values calculated by different models and the experimental stress values. (**a**,**b**) $\dot{\varepsilon }$ = 0.01 s^−1^; (**c**,**d**) $\dot{\varepsilon }$ = 0.1 s^−1^; (**e**,**f**) $\dot{\varepsilon }$ = 1 s^−1^.

The predicted values of the constitutive models established in the previous section were compared to the experimental values. The results are depicted in Figure 10. For the A-T model, the optimization effect was evident at 800 °C. For the JC model, the optimization effect was more pronounced under the three deformation conditions of 900 °C/1 s^−1^, 950 °C/1 s^−1^, and 1000 °C/1 s^−1^, as depicted in Figure 10f. Furthermore, since 800 °C/0.01 s^−1^ was the reference condition of the JC model, therefore the JC model exhibited higher predictive reliability than the A-T model under this deformation condition.

To quantitatively assess the reliability of the above two models, R and AARE were introduced. Their formulas can be expressed as:

(14) $$R=\frac{\sum_{i=1}^{N}\left(\sigma_{e}^{i}-{\bar{\sigma }}_{e}\right)\left(\sigma_{cal}^{i}-{\bar{\sigma }}_{cal}\right)}{\sqrt{\sum_{i=1}^{N}{\left(\sigma_{e}^{i}-{\bar{\sigma }}_{e}\right)}^{2}}\sqrt{\sum_{i=1}^{N}{\left(\sigma_{cal}^{i}-{\bar{\sigma }}_{cal}\right)}^{2}}}$$

(15) $$AARE=\frac{1}{N}\overset{N}{\underset{i=1}{\sum }}\left|\frac{\sigma_{e}^{i}-\sigma_{cal}^{i}}{\sigma_{e}^{i}}\right|\times 100\%$$

In the formula, $\sigma_{e}$ is the experimentally measured stress, $\sigma_{\mathrm{cal}}$ is the stress calculated by the model, ${\bar{\sigma }}_{e}$ is the average of all $\sigma_{e}$ measured in the experiment, and ${\bar{\sigma }}_{cal}$ is the average of all $\sigma_{cal}$.

The value of R indicates the degree of correlation between the predicted values and the experimental values. However, because R does not consider bias, higher R values do not always mean a better predictiveness of the constitutive model. AARE is an unbiased statistical parameter used to measure the predictiveness of a model or equation. The smaller the AARE value, the higher the predictiveness of the model.

**Figure 11:**
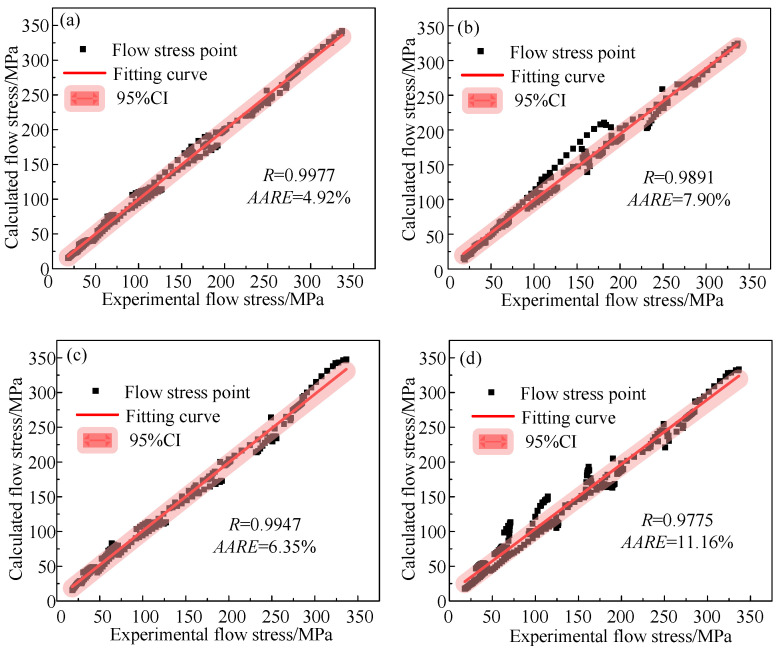
The correlation between the calculated values from the established models and the experimental values of hot compression: (**a**) optimized A-T model; (**b**) unoptimized A-T model; (**c**) optimized JC model; and (**d**) unoptimized JC model.

It can be seen from Figure 11 that the optimized A-T model has an R value of 0.9977 and an AARE value of 4.92%. The R value of the optimized A-T model is 0.0086 greater than that of the unoptimized A-T model, and the AARE value of the optimized A-T model is 2.98% lower than that of the unoptimized A-T model. The optimized JC model has an R value of 0.9947 and an AARE value of 6.35%. Similarly, the optimized JC model showed an increase in R value and a decrease in AARE value, with an increase of 0.0172 and a decrease of 4.81%, respectively. This shows that the proposed parameter inverse optimization identification method is feasible.

As shown in Figure 11a,c, after optimization, both the A-T model and the JC model had high predictability for the Ti6Al4V alloy. After comparing the A-T model and the JC model, it was found that the R value of the former was 0.003 higher than that of the latter, while the AARE value of the former was 1.43% lower than that of the latter. It was shown that, after optimization, the A-T model was better suited than the JC model for describing the high-temperature deformation behavior of the Ti6Al4V Alloy.

For the purpose of further assessing the prediction accuracy of the two optimized models, the RE between experimental stress and model-calculated stress was analyzed. The formula for RE is as follows:

(16) $$RE=\frac{\sigma_{e}^{i}-\sigma_{cal}^{i}}{\sigma_{e}^{i}}\times 100\%$$

In the formula, the definitions of $\sigma_{e}$ and $\sigma_{cal}$ are the same as in the previous formulas.

**Figure 12:**
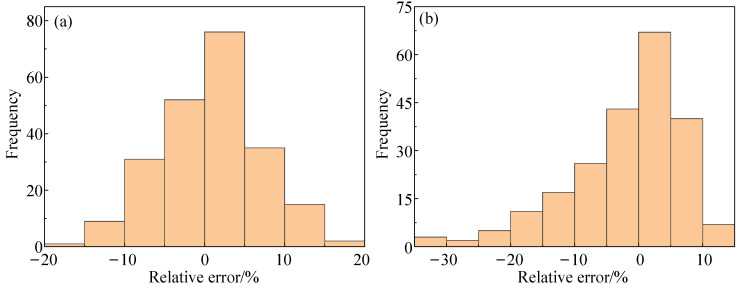
Distribution diagram of relative errors between the two optimized models: (**a**) the optimized A-T model; (**b**) the optimized JC model.

Figure 12 shows the RE distributions of the optimized A-T model and the optimized JC model. It can be seen from Figure 12 that the RE fluctuation range of the optimized A-T model was between −20% and 20%, while the maximum RE of the optimized JC model reached −35%. The mathematical expectation and standard deviation of the optimized A-T model RE were 0.67406 and 6.34041, respectively, and those of the optimized JC model were −2.36206 and 9.77954, respectively, as determined by calculation. This indicates that the RE distribution of the optimized A-T model was more concentrated and tended to a smaller RE value. This further confirms that the A-T model had a greater predictive accuracy compared to the JC model.

## 5. Conclusions

In this paper, the thermal deformation behavior of Ti6Al4V alloy was analyzed. The A-T model and the JC model were, respectively, established by using the parameter inverse optimization identification method. Finally, the accuracy of the two models’ predictions was quantitatively compared by introducing standard statistical parameters. The specific conclusions obtained are:

(1) The true stress of Ti6Al4V alloy increases with the increase of strain rate and decreases with the increase of temperature. Below 850 °C, the Ti6Al4V alloy will show obvious dynamic recrystallization characteristics. Since the α phase will gradually transform into the β phase above 850 °C, and the β phase has high stacking fault energy, the flow stress shows a dynamic recovery phenomenon above 950 °C.
(2) The A-T model and JC model established by the inverse optimization identification method both exhibit higher R values and smaller AARE values, demonstrating the feasibility of the method proposed in this paper.
(3) After parameter inverse optimization, both the A-T constitutive model and the JC constitutive model showed high prediction accuracy. In contrast, the optimized A-T model exhibited higher R values and lower AARE values. And the distribution of the A-T model RE is more concentrated and tends to a smaller value. This shows that the optimized A-T constitutive model is more suitable for describing the high-temperature deformation behavior of the Ti6Al4V alloy.

## Figures and Tables

**Table 1:** Chemical composition of the Ti6Al4V alloy (mass percentage: wt.%).

Ti	Al	V	Fe	O	C	N	H
Bal.	6.11	3.93	0.131	0.113	0.016	<0.005	<0.001

**Table 2:** Description of each character in Formulas (6)–(8).

Parameters	Description
*R*	Universal gas constant (8.314 J·mol^−1^·K^−1^)
*T*	Absolute temperature (*K*)
*Q*	Activation energy (J·mol^−1^)
$\dot{\varepsilon }$	Strain rate (s^−1^)
σ	True stress (MPa)
*A*, *n*, *α*, *β*, and n′	Material parameters

**Table 3:** Material parameters for various strains determined using the regression method.

True Strain	lnA	*n*	α (MPa^−1^)	*Q* (J·mol^−1^)
0.05	62.59874	3.997360	0.010820	651,391.9
0.10	60.44771	3.839814	0.010609	630,040.1
0.15	58.22757	3.730126	0.010536	607,614.3
0.20	55.38215	3.598379	0.010586	578,947.0
0.25	52.00925	3.447356	0.010697	544,882.9
0.30	48.65197	3.298253	0.010841	510,931.3
0.35	45.41233	3.152317	0.010990	478,161.1
0.40	42.57605	3.014204	0.011182	449,493.6
0.45	40.12852	2.890635	0.011393	424,775.1
0.50	38.16642	2.772139	0.011700	405,096.2
0.55	36.94857	2.679515	0.012090	393,049.2
0.60	36.20739	2.605249	0.012536	385,921.8
0.65	35.72397	2.547709	0.012989	381,367.5

**Table 4:** Material parameters under different strains obtained by the parameter inverse optimization identification method.

True Strain	lnA	*n*	α (MPa^−1^)	*Q* (J·mol^−1^)
0.05	58.12871	4.23524	0.00779	587,663.1875
0.10	57.33795	4.28375	0.00696	574,962.7500
0.15	55.88721	4.21043	0.00687	560,393.4375
0.20	53.43388	4.07124	0.00694	536,722.6250
0.25	50.26610	3.88292	0.00714	506,615.8125
0.30	46.97851	3.68291	0.00741	475,486.5000
0.35	43.79500	3.48414	0.00771	445,391.3750
0.40	40.98165	3.29625	0.00802	418,717.09375
0.45	38.53254	3.12380	0.00837	395,618.4375
0.50	36.47949	2.98140	0.00862	375,677.59375
0.55	35.02670	2.87397	0.00879	361,163
0.60	33.98806	2.80240	0.00878	349,939.09375
0.65	33.11206	2.77945	0.00851	339,260.21875

**Table 5:** Parameter values for the unoptimized A-T model.

Polynomial Order	α(ε)	n(ε)	Q(ε)	lnA(ε)
0	0.01121	4.24446	673,570.20699	64.88817
1	−0.00938	−6.71438	−530,642.20922	−55.43134
2	0.02978	44.36086	2,495,163.15361	272.19777
3	0.08099	−215.56526	−1.91346 × 10^7^	−2008.70089
4	−0.53248	506.28031	5.01289 × 10^7^	5245.6406
5	0.94031	−569.67117	−5.47281 × 10^7^	−5772.14165
6	−0.54365	249.93973	2.19473 × 10^7^	2345.23846

**Table 6:** Parameter values for the optimized A-T model.

Polynomial Order	α(ε)	n(ε)	Q(ε)	lnA(ε)
0	0.00985	4.00538	601,356.55463	58.22586
1	−0.06046	6.93251	−391,942.19605	−1.45882
2	0.4586	−52.6009	3,447,384.32426	51.5667
3	−1.717	126.12082	−2.73235 × 10^7^	−1642.34849
4	3.55327	−166.1522	7.46826 × 10^7^	5241.01491
5	−3.75706	124.05828	−8.76861 × 10^7^	−6406.99498
6	1.55652	−38.17755	3.82251 × 10^7^	2823.42712

**Table 7:** Description of parameters in the JC model.

Parameters	Description	Parameters	Description
$T^{*}$	$\frac{T-T_{ref}}{T_{m}-T_{ref}}$	${\dot{\varepsilon }}^{*}$	$\frac{\dot{\varepsilon }}{{\dot{\varepsilon }}_{ref}}$
$T_{ref}$	Reference temperature (°C)	${\dot{\varepsilon }}_{ref}$	Reference strain rate (s^−1^)
σ	True stress (MPa)	ε	True strain
*T*	Temperature (°C)	$\dot{\varepsilon }$	Strain rate (s^−1^)
$T_{m}$	Melting point of Ti6Al4V alloy	*A*(ε), *B*(ε), *C*(ε), *D*(ε)	Third-order polynomial functions on strain
*Ai*, *Bi*, *Ci*, *Di* (*i* = 0, 1, 2, 3)	Polynomial coefficients		

**Table 8:** The values of third-order polynomial coefficients for the unoptimized JC model.

Polynomial Order	A(ε)	B(ε)	C(ε)	D(ε)
0	183.52348	0.1621	−8.52844	0.26029
1	−20.72312	0.25494	2.8761	1.85946
2	−411.73526	0.09378	−3.67844	−0.78508
3	401.93831	0.00401	1.8396	−1.6864

**Table 9:** The coefficient value of the third-order polynomial obtained by the parameter inverse optimization identification method.

Polynomial Order	A(ε)	B(ε)	C(ε)	D(ε)
0	186.51511	0.17328	−8.124	0.24784
1	13.67602	0.17068	2.19076	0.0565
2	−481.34012	0.24383	−3.65103	0.7116
3	420.43158	−0.04438	0.01	0.1

## Data Availability

The data presented in this study are available on request from the corresponding author. The data are not publicly available due to these data being part of ongoing research.
